# Evaluating blinding in trials of endometrial scratching: a randomised nested survey within two randomised controlled trials

**DOI:** 10.1186/s13063-026-09763-8

**Published:** 2026-07-15

**Authors:** Sarah Lensen, Afsana Afroz, Lynn Sadler, Cindy Farquhar, Jack Wilkinson

**Affiliations:** 1 Department of Obstetrics, Gynaecology and Newborn Health, University of Melbourne, Royal Women’s Hospital, Melbourne, Australia; 2 Department of Obstetrics and Gynaecology, University of Auckland, Auckland, New Zealand; 3 Centre for Biostatistics, School of Health Sciences, Faculty of Biology, Medicine and Health, Manchester Academic Health Science Centre (MAHSC), University of Manchester, Manchester, UK

**Keywords:** Endometrial scratching, Blinding, Blinding index, Placebo, Sham, Performance bias

## Abstract

**Background:**

Blinding of participants in randomised controlled trials is considered a robust method to protect against biases associated with departures from the intended intervention and measurement of the outcome. Even when implemented, the success of blinding is not guaranteed, and participants may be able to discern their allocation through the experience of medication side effects or procedural discomfort. To assess the effectiveness of blinding, formal evaluation tools such as the James and Bang blinding indices have been developed.

**Aim:**

To explore whether participants can be effectively blinded in randomised controlled trials involving physical procedures, using endometrial scratching as a case study. Secondary aims were to measure whether symptoms from the procedures (pain, bleeding, and pregnancy outcome) are associated with unblinding, and whether behaviours of trial participants (intercourse patterns and protocol violations) may manifest as a result of unblinding.

**Methods:**

A randomised survey was conducted during two existing trials of endometrial scratching compared to a placebo procedure, in couples attempting to conceive from intercourse. Participants were asked to guess their trial allocation and to provide a reason for their guess. Participants were randomised to guess their allocation either early (within 1 week of study intervention) or late (after their window to conceive had closed). Blinding was assessed using both Bang’s and James’ blinding indexes, and the association of participant guesses and the measured symptoms and behaviours was evaluated by chi-squared test and the independent Student’s *t*-test.

**Results:**

Of 278 participants, 56% correctly guessed their allocation, 32% guessed incorrectly, and 21% selected ‘don’t know’. Pain and bleeding were significantly higher in the endometrial scratching arm and were associated with correct guessing. Blinding indices suggested a lack of blinding, particularly in the intervention group (James’ blinding index 0.42 (0.36–0.48)). No differences were observed in intercourse timing or protocol violations between groups.

**Conclusion:**

The sham procedure used in these studies does not appear to have achieved complete blinding; however, there was no evidence that lack of blinding led to impacts on important trial behaviour, such as frequency of intercourse.

**Trial registration:**

ACTRN12614000657628, ACTRN12614000656639. Registered on June 24 2014.

**Supplementary Information:**

The online version contains supplementary material available at https://doi.org/10.1186/s13063-026-09763-8.

## Background

Randomisation is the most effective method for preventing confounding and ensuring comparability between treatment groups. However, randomisation alone cannot protect against all forms of bias. One threat to trial validity is performance bias, which arises when knowledge of treatment allocation influences the behaviour of trial participants or personnel in a manner that differs in the trial context compared to real-world use of the intervention. In the absence of blinding, the trial context can lead to systematic differences in care or use of adjunct interventions, ultimately distorting treatment effects and compromising the reliability of trial findings [1]. A common method to minimise performance bias is to blind or mask participants and study personnel to trial allocation, for example with the use of a placebo or sham.

Empirical evidence from meta-epidemiological studies is mixed as to whether unblinded trials are more likely to report exaggerated treatment effects, thereby providing misleading information about intervention benefits [2–6]. Trials including randomised sub-cohorts of blinded and non-blinded participants also demonstrate inflated treatment effects among the non-blinded cohorts [7].

Despite the advantages of blinding, its implementation remains inconsistent. An audit of RCTs published in leading medical journals found that approximately 40% of RCTs are reported as double-blind, the remainder being partially or completely open-label [8]. Several factors likely contribute to this observation, including the logistical challenges of creating a suitable placebo, the cost of placebo manufacturing, and the perception that blinding is unnecessary in studies with objective endpoints such as death [9]. Furthermore, some researchers believe that blinding increases recruitment difficulties, and that participants may feel deceived or distrustful of trial personnel, which could lead to participant withdrawal or protocol violations, which in turn affect the reliability of the trial results [10, 11].

Even when blinding is employed, its effectiveness is not guaranteed. Blinding can be compromised if active and placebo interventions are distinguishable by appearance, taste, or side effects. As a result, some trials may be described as double-blinded even when participants were able to discern their allocation. One approach to assessing blinding effectiveness is to ask participants to guess their treatment allocation [12, 13]. However, the optimal timing for such assessments remains controversial. Early assessments may fail to detect unblinding that occurs at a later time, whereas late assessments may be influenced by treatment outcomes [13–17]. Some researchers advocate for multiple assessments throughout the trial to evaluate changes in blinding status over time [18, 19], though repeated questioning may itself encourage participants to attempt to uncover their allocation [20].

To quantify the success of blinding, two indices have been developed: James’ blinding index (JBI) and Bang’s blinding index (BBI) [21, 22]. JBI appropriately assigns greater weight to participants who respond ‘don’t know’, reflecting the assumption that successful blinding results in uncertainty about allocation [19]. However, JBI combines responses from both trial arms, limiting its ability to detect differential unblinding between groups. In contrast, BBI is more sensitive to unblinding and allows for separate assessment within each treatment arm [22]. While both indices have strengths and limitations, using them in combination may provide a more comprehensive assessment of blinding integrity [19].

**Fig. 1:**
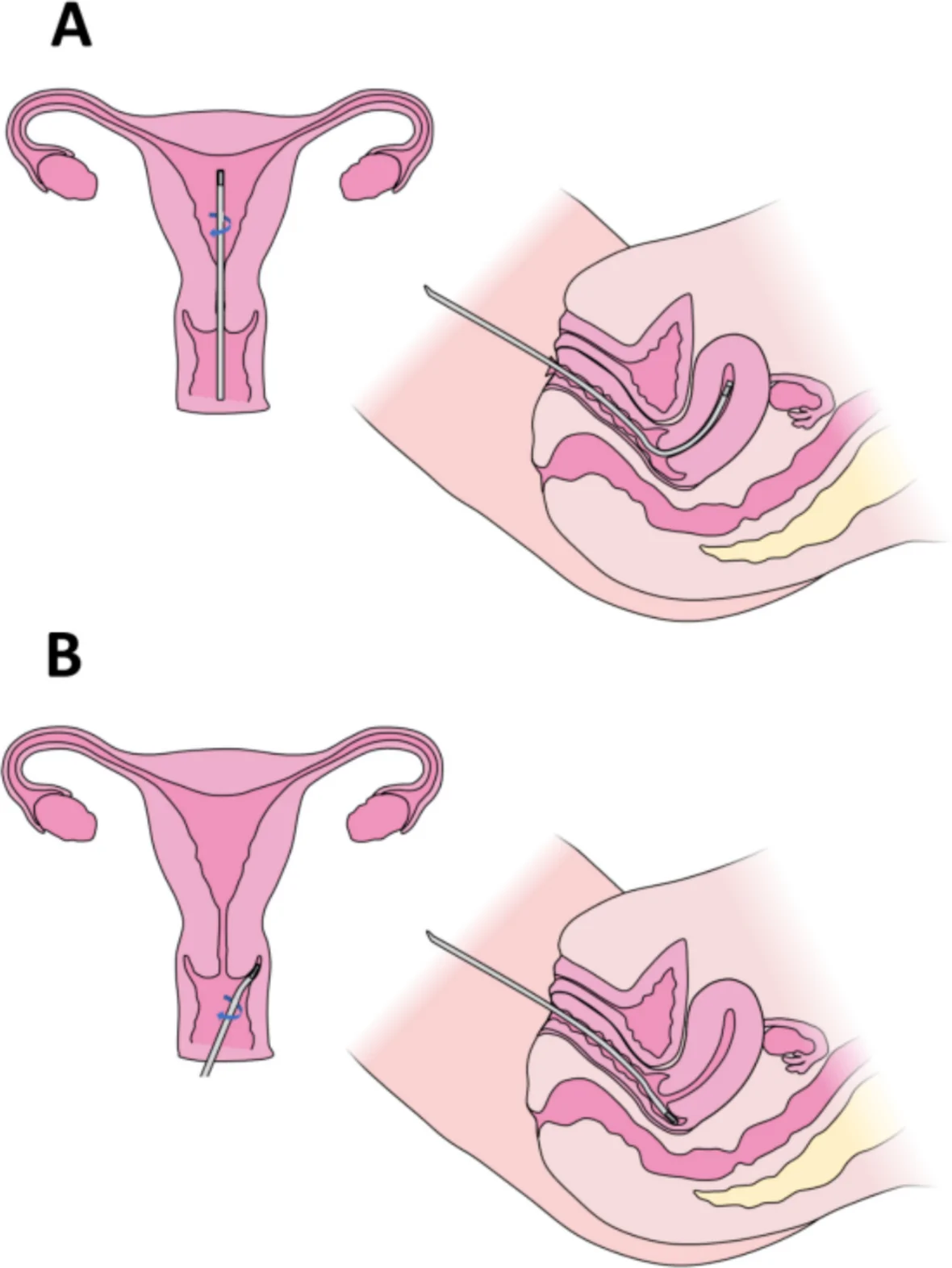
Cross-sectional diagram of intervention and sham procedures. **A** A depiction of a standard pipelle biopsy/endometrial scratch procedure, with placement of the pipelle inside the womb in order to obtain an endometrial biopsy. **B** A depiction of the sham procedure used, with placement of the pipelle in the posterior fornix, with no insertion of the instrument into the external os of the cervix. The internal piston is withdrawn and the catheter moved and rotated to replicate the standard biopsy action. Image created by Matthew McAlpine

This study reports on the assessment of blinding in two similar trials of endometrial scratching among subfertile couples attempting to conceive from intercourse [23, 24]. An endometrial scratch procedure involves placement of a thin plastic pipelle through the cervix and into the uterus to obtain a sample of endometrial tissue through the mechanical action of suction and rotation (Fig. 1A). These trials utilised a sham procedure in which the pipelle was positioned in the posterior fornix of the vagina, without inserting it into the cervix or uterine cavity (Fig. 1B). While this approach minimised potential contamination or incidental ‘scratching’ of the cervix or uterus, it also risked failing to fully blind participants due to likely differences in discomfort levels. Given that most participants had no prior experience with endometrial biopsy, their expectations regarding pain were expected to be varied.

## Aims

This randomised survey aimed to investigate the effectiveness of a sham procedure for endometrial scratching. Participants from two existing trials of endometrial scratching compared to a sham, followed by regular unprotected intercourse, were randomised to guess their study allocation at either an early or late stage of the trial.

There were the following three specific aims:

1. Does the use of a placebo procedure blind participants to their treatment allocation?
2. Do symptoms or side effects from the procedure (real or perceived) influence participant allocation guessing, specifically pain during procedure, bleeding following procedure, and pregnancy outcome?
3. Do measured behaviours indicate success of blinding, specifically episodes of intercourse for three cycles and occurrence of protocol violations?

## Methods

### Setting

Participants were taking part in one of two ongoing trials comparing endometrial scratching to a sham procedure [23, 24]. All participants were scheduled for a clinic visit between days 1 and 12 of a menstrual or ovulation induction cycle. They were advised to arrive with a partly filled bladder and to take paracetamol 60 min prior. Upon arrival, participants were randomised to either the endometrial scratching or sham groups, and informed that the procedure could be uncomfortable, with varying experiences among individuals. A web-based external system, InferMed Macro (InferMed UK), was employed for both data collection and randomisation, enabling verification of participant eligibility and maintaining allocation concealment up to the moment of assignment. Participants were randomised in a 1:1 ratio using a block design with variable block sizes (8 and 10), applied in a randomly alternating sequence and stratified by study site. Following randomisation, participants remained blinded to their assigned group, whereas the clinician performing the intervention was unavoidably aware of the allocation. Clinicians maintained blinding by ensuring both procedures took a similar length of time, using consistent instructions, and employing distraction techniques for all patients, such as asking participants to cough as the pipelle is passed through the cervix.

Immediately after the procedure, participants recorded their pain on a 10-cm VAS scale (0 = no pain, 10 = worst pain) and were provided with a study diary to log bleeding the day after the procedure, menstruation dates, and episodes of intercourse for three menstrual or ovulation induction cycles. Participants attempted to conceive via intercourse in their current cycle and for two subsequent cycles. During the trial, if pregnancy was suspected, participants was advised to take a home test. Participants with positive pregnancy tests contacted the coordinator for a viability scan at 6–8 weeks’ gestation, and all pregnancies were followed to live birth.

### Randomisation and blinding

Within 2 days of the study procedure, participants were randomly allocated to guess their study allocation either early or late. The same web-based system, InferMed Macro, was employed for randomisation as described above, which enabled allocation concealment up to the moment of assignment. Participants were randomised in a 1:1 ratio using a block design with variable block sizes (8 and 10), applied in a randomly alternating sequence and stratified by study site. Early corresponded to a guess within the first 2 weeks of the trial, and late corresponded to a guess at the end of the 3-month study period (when the participant knew whether or not they had fallen pregnant). Participants were contacted by email or phone and asked to guess their allocation using a 2 × 3 format with the options of ‘don’t know’, ‘endometrial scratching’, and ‘placebo’ [25]. Participants were also asked to provide a reason for their guess as an open-ended question. Reasons were then grouped into the following categories: pain from procedure, use of extra instruments during procedure, bleeding after procedure, attitude of personnel, hunch, pregnancy outcome, other. Participants randomised to early were also asked to guess again at the end of the study (late). Participants were informed of their allocation after providing their late guess.

### Statistical analysis

Assessment of participant blinding was undertaken using both James’ and Bang’s blinding indexes [21, 22]. Missing data were handled using a complete case approach, and no imputation was applied. An intercourse score for each cycle was determined from the study diary as follows: a high score if at least one episode of intercourse occurred between 10 and 13 days prior to the onset of the next menses (the most fertile period); medium if at least one episode occurred 9, 14, or 15 days prior to the subsequent menses; and low if no intercourse occurred between 9 and 15 days prior to the next menses. For example, if a participant with a 28-day cycle had intercourse on cycle days 7, 11, and 15, they would be assigned an intercourse score for that cycle of ‘high’. The overall score for each participant was the most common score across the three cycles, with ‘high’ being assigned if the participant had one each of low, medium, and high scoring cycles.

Women were defined as having a protocol violation if they were:

1. Lost to follow-up
2. Misplaced or did not return the study diary
3. Had at least one cycle with no episodes of intercourse
4. Were allocated to the early group and did not supply the late guess
5. Committed any other protocol violation such as undergoing any additional intrauterine procedure, or any fertility treatment such as intrauterine insemination or in vitro fertilisation (IVF)

The chi-squared test was used to measure associations between categorical variables, such as trial allocation and occurrence of protocol violations. The independent Student’s *t*-test was used to measure differences in means between groups, for example mean pain scores. These analyses were undertaken in two ways. The first analysis compared the endometrial scratching group to the placebo group (ignoring guesses). This replicated the information available to most studies as participants are seldom requested to guess their allocation in trials [12, 13]. The second set of analyses assessed participants based on their guesses. These analyses were undertaken separately in women randomised to the endometrial scratching and placebo groups, to control for confounding from actual trial allocation. Participants randomised to guess early were also asked to guess late; however, only their early guesses were used in these analyses. The late guesses from these participants were only used for the assessment of whether there were changes in guessing between early and late. The blinding indices were calculated using the BI package in R (R Core Team, 2024). All other analyses were undertaken using Stata V.17 (Stata-Corp., College Station, TX, USA). Any *p* value < 0.05 was considered to be statistically significant. No power calculation was undertaken specific to this research as the sample size calculations were undertaken for the primary outcome of the two individual trials [23, 24]. The statistician performing the data analysis was not blind to the study allocations.

## Results

### Participants

**Fig. 2:**
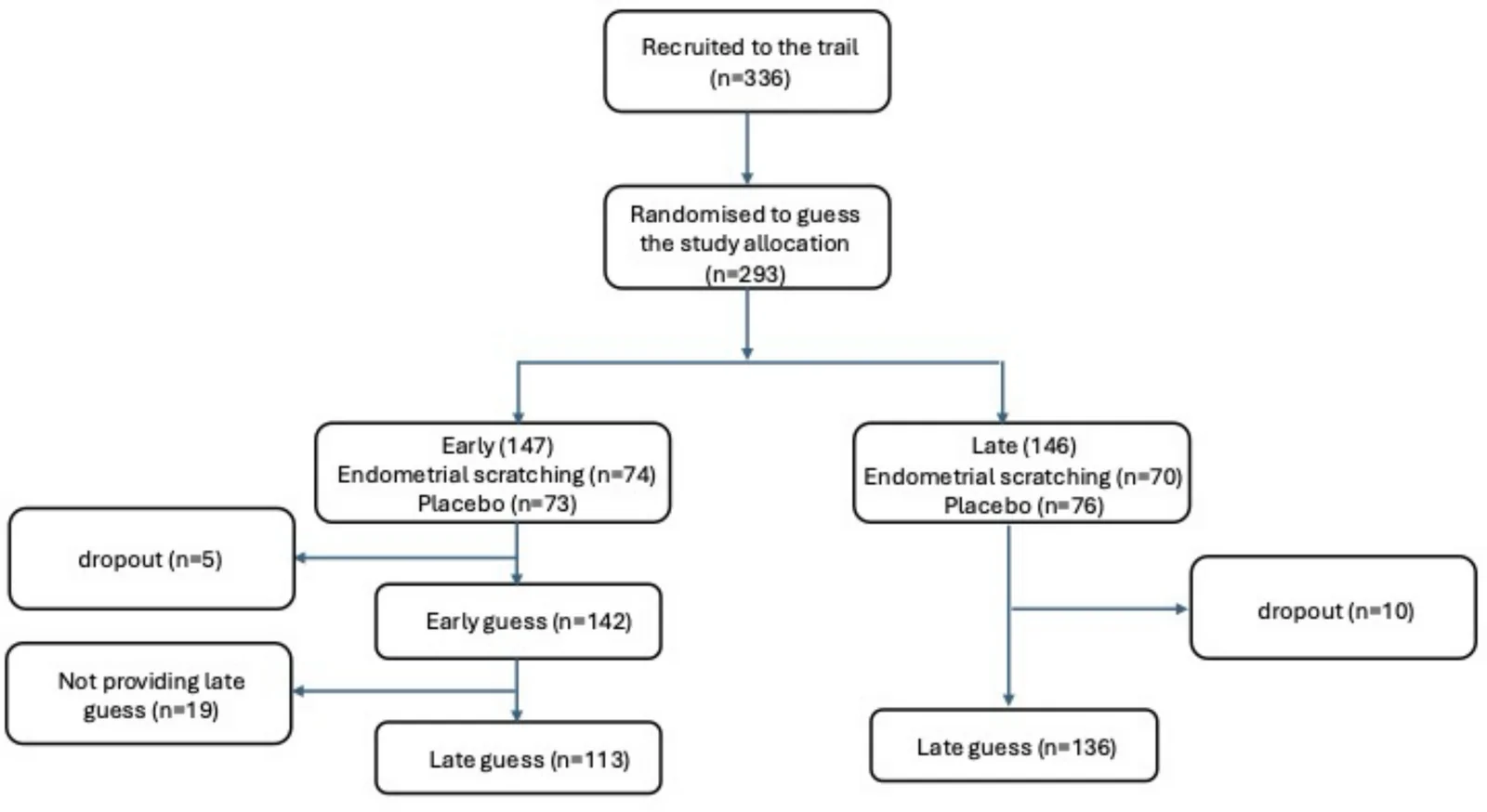
Study flow diagram

A total of 336 women were included in the existing trials, and 293 were randomised to guessing their study allocation, as guessing trial allocation was introduced 6 months following trial initiation. Of the 293 randomised women, 278 were included in the analysis after excluding 15 ineligible participants who did not provide at least one guess (Fig. 2). Of the 278 included participants, 139 women were randomised to endometrial scratching and 139 to placebo, and 142 were allocated to guess early, and 136 to guess late. Additionally, 113 of those allocated to guess early also provided guesses late.

### Assessment of blinding

In the endometrial scratching arm, 32 (23%) women guessed ‘don’t know’, 89 (64%) correctly guessed allocation to the endometrial scratching arm, and 18 (13%) guessed allocation to the placebo arm (Table 1). In the placebo group, 27 (19%) guessed ‘don’t know’, 72 (52%) correctly guessed allocation to the placebo arm, and 40 (29%) guessed allocation to the endometrial scratching arm. In total, 59/278 (21%) guessed ‘don’t know’, 129/278 (56%) women guessed their correct allocation, and 90/278 (32%) guessed incorrectly.

**Table 1:** Participant guesses of trial allocation, by true trial allocation and by timing of guesses, including James’ and Bangs’ blinding indexes

	Guessed scratching *N* (%)	Guessed placebo *N* (%)	Don’t know *N* (%)	James’ blinding index (95% CI)	Bang’s blinding index (95% CI)
All guesses (*N* = 278)					
Scratching	89 (64.0)	18 (13.0)	32 (23.0)	0.42 (0.36 to 0.48)	0.51 (0.39 to 0.63)
Placebo	40 (28.8)	72 (51.8)	27 (19.4)		0.23 (0.09 to 0.37)
Early (*N* = 142)					
Scratching	51 (69.9)	7 (9.6)	15 (20.5)	0.41 (0.33 to 0.49)	0.60 (0.45 to 0.75)
Placebo	20 (29.0)	33 (47.8)	16 (23.2)		0.19 (−0.01 to 0.39)
Late (*N* = 136)					
Scratching	38 (57.8)	11 (16.7)	17 (25.8)	0.43 (0.35 to 0.51)	0.41 (0.23 to 0.59)
Placebo	20 (28.6)	39 (55.7)	11 (15.7)		0.27 (0.07 to 0.48)

Interpretation: James’ index ranges from 0 to 1: a score of 0 indicates complete unblinding, 0.5 indicates random guessing/successful blinding, and a score of 1 indicates all participants guess the opposite of their true allocation. Unblinding may be claimed if the two-sided confidence interval does not cover 0.5 (i.e. if the upper bound of the interval is below 0.5); Bang’s index ranges from −1 to 1: a score of −1 indicates all participants guessing the opposite of their true allocation, 0 indicates random guessing/successful blinding, and 1 indicates complete unblinding. Unblinding may be claimed if the confidence interval does not cover 0

*CI* confidence interval

Success of blinding was examined using both James’ and Bang’s blinding indexes (Table 1). Overall, James’ index suggested a lack of blinding as the confidence interval approached but did not cross 0.5—except for the late guesses (Table 1). Bang’s blinding index also demonstrated evidence of unblinding among all participants with greater unblinding suggested within the scratching arm.

**Fig. 3:**
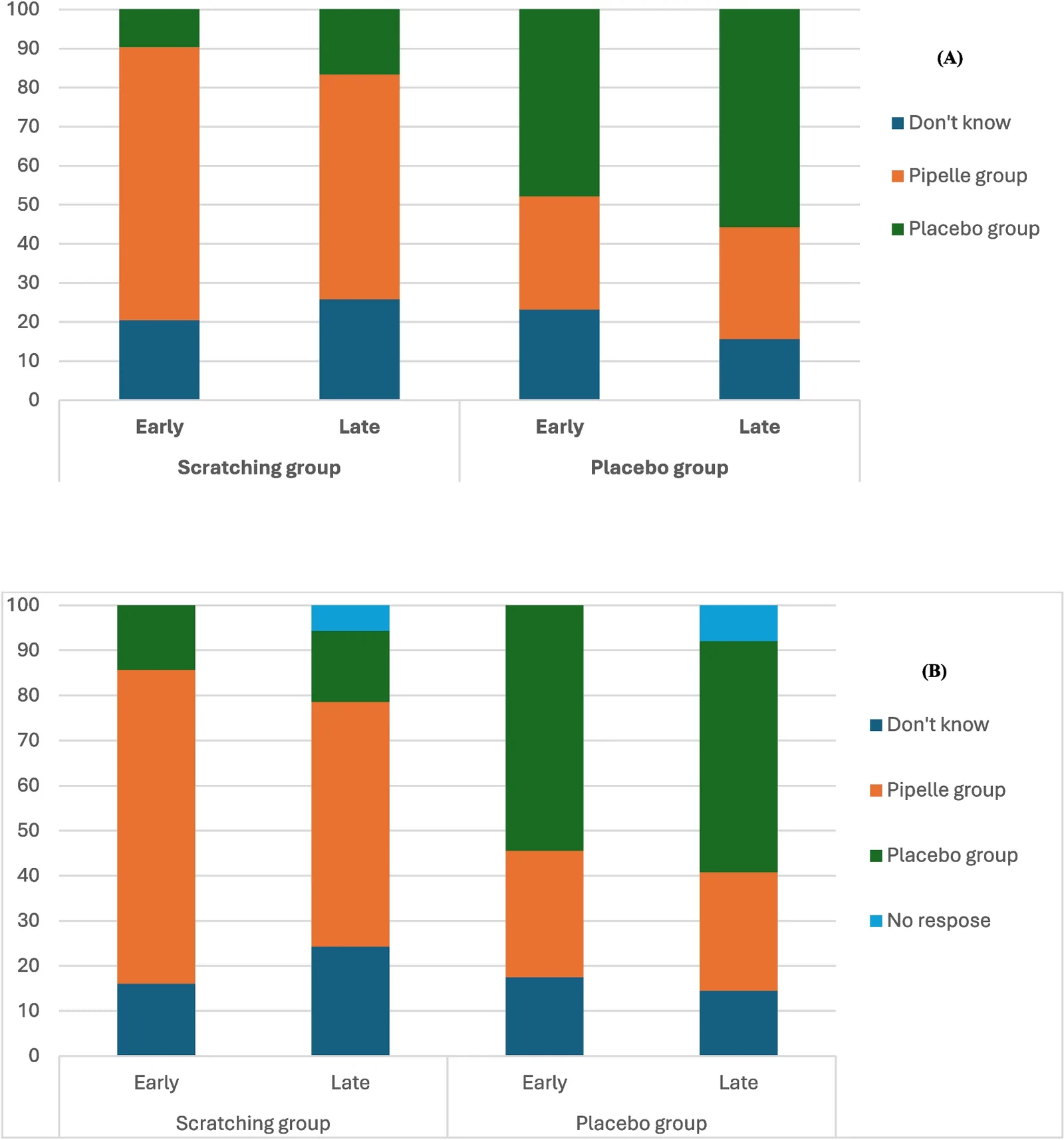
Guessing of trial allocations among survey participants. **A** Allocation guesses in all participants, by trial allocation. **B** Response changes over time among those allocated to guessing early, by trial allocation

There appear to be minor differences in unblinding between participants guessing early and those guessing late. Participants guessing late were more likely to guess they were allocated to the placebo group; however, this was not statistically significant (*p* = 0.277 in endometrial scratching group, *p* = 0.492 in placebo group) (Fig. 3A, Table 1). Participants randomised to guess early were also asked to guess their allocation a second time (late), allowing an opportunity to measure changes in belief over time. The distribution of guesses was similar at the two time points; indeed, most participants (75%, 85/113) did not change their guess over time (Fig. 3B).

### Symptoms

There are three measurable symptoms or effects of the scratching and placebo procedures which may lead to (perceived) unblinding of participants: pain experienced during the procedure, bleeding following the procedure, and pregnancy outcome in the trial.

#### Pain during procedure

Women were asked to record the level of pain they experienced during the procedure on a 10-cm VAS. The mean pain score was significantly higher in the scratching arm than in the placebo group (mean score 4.5 ± 2.4 vs. 1.4 ± 1.5, *p* < 0.001) (Table 2).

**Table 2:** Symptoms and behaviours of trial participants, and associations with trial allocation and with allocation guesses

	Scratching					Placebo					*p* value (comparing scratch vs. placebo)
	Total (*n* = 139)	Guess scratch (*n* = 89)	Guess placebo (*n* = 18)	Don’t know (*n* = 32)	*p* value (comparing guess)	Total (*n* = 139)	Guess scratch (*n* = 40)	Guess placebo (*n* = 72)	Don’t know (*n* = 27)	*p* value (comparing guess)	
Symptoms											
Pain (mean ± SD)	4.5 ± 2.4					1.4 ± 1.5					<0.001
Pain score < mean		32 (45.7)	15 (21.4)	23 (32.9)	<0.001		19 (33.9)	25 (44.6)	12 (21.4)	0.370	
Pain score > mean		57 (82.6)	3 (4.4)	9 (13.0)			21 (25.3)	47 (56.6)	15 (18.1)		
Missing (*n*)	0					4					
Bleeding											
Bleeding	87 (67.4)	59 (67.8)	17 (19.5)	17 (19.5)	0.383	20 (16.3)	10 (50.0)	5 (25.0)	5 (25.0)	0.006	<0.001
No bleeding	42 (32.6)	24 (57.1)	7 (16.7)	11 (26.2)		103 (83.7)	25 (24.3)	62 (60.2)	16 (15.5)		
Missing (*n*)	10					14					
Pregnancy outcome (only late guess, *n* = 136)											
Not pregnant	57 (86.4)	29 (50.9)	11 (19.3)	17 (29.8)	0.022	62 (88.6)	15 (24.2)	38 (61.3)	9 (14.5)	0.028	0.490
Pregnant	9 (13.6)	9 (100.0)	0 (0.0)	0 (0.0)		8 (11.4)	5 (62.5)	1 (12.5)	2 (25.0)		
Behaviours											
Intercourse score											
Low	1 (0.8)	0 (0.0)	0 (0.0)	1 (3.2)	0.079	5 (3.9)	4 (11.1)	1 (1.4)	0 (0.0)	0.073	0.359
Medium	13 (10.2)	7 (8.5)	0 (0.0)	6 (19.4)		9 (7.1)	4 (11.1)	4 (5.8)	1 (4.5)		
High	115 (89.1)	75 (91.5)	16 (100)	24 (77.4)		113 (89.0)	28 (77.8)	64 (92.8)	21 (95.5)		
Missing (*n*)	10					12					
Protocol violations											
Yes	24 (17.3)	17 (19.1)	5 (27.8)	2 (6.3)	0.115	23 (16.5)	7 (17.5)	11 (15.3)	5 (18.5)	0.911	0.501
No	115 (82.7)	72 (80.9)	13 (72.2)	30 (93.8)		116 (83.5)	33 (82.5)	61 (84.7)	22 (81.5)		
Missing (*n*)	0					0					

Denominators used were the total number of responses/measures for each variable (excluding missing data). For the symptoms, denominators were the number of women in each symptom subgroup, for example the number of women with a pain score above average, to address the question ‘Are women with a mean pain score above average more likely to guess allocation to the endometrial scratching group?’ For the behaviours, denominators were the totals for the guessing subgroups, for example the number of women guessing allocation to the placebo arm, to address the question ‘Are women guessing allocation to the placebo arm, more likely to commit a protocol violation?’

Within the endometrial scratching group, women with a mean pain score above average were more likely to guess they were allocated to the endometrial scratching arm than women with a pain score below average (*p* < 0.001). However, the same trend was not observed in the placebo arm.

#### Bleeding after procedure

Women were asked to report whether they had any vaginal bleeding the day following the procedure (excluding menstruation). Women in the endometrial scratching group were more likely to experience bleeding (67%) than women in the placebo group (16%) (*p* < 0.001) (Table 2).

Among women in the endometrial scratching group, those who experienced bleeding were more likely to correctly guess their allocation; however, this was not statistically significant (*p* = 0.383). Among women in the placebo arm, those experiencing bleeding were more likely to guess allocation to endometrial scratching than those not experiencing bleeding (*p* = 0.006).

#### Pregnancy outcome

Across both studies, there was no evidence of any impact of endometrial scratching on the chance of pregnancy [23, 24]. Pregnancy outcomes were only relevant for participants guessing late, as those guessing early did not yet know if they had conceived during the study. In the scratching group, all women who became pregnant (*n* = 9) correctly guessed they were in the scratching arm, and most women who conceived in the placebo group also guessed they were in the scratch arm (63%).

### Reasons supporting participant guessing

Participants were asked to provide a free-text reason to support their choice of guess. In the endometrial scratching arm, those correctly guessing their allocation was endometrial scratching most often reported the reason as pain during the procedure (64%) (Table 3). Similarly, women in the placebo arm guessing their allocation was placebo were more likely to report (lack of) pain as the reason.

**Table 3:** Reasons supporting participant guesses, by guesses and by trial allocation

Symptoms	Scratch (*N* = 139)			Placebo (*N* = 139)		
	Guess scratch (*n* = 83) *N* (%)	Guess placebo (*n* = 17) *N* (%)	Don’t know (*n* = 17)	Guess scratch (*n* = 37) *N* (%)	Guess placebo (*n* = 70) *N* (%)	Don’t know (*n* = 9)
Pain during procedure	53 (64)	4 (24)	6 (35)	16 (43)	25 (36)	4 (44)
Use of extra instruments	0 (0.0)	0 (0.0)	0 (0.0)	1 (2.7)	0 (0.0)	0 (0.0
Bleeding after procedure	8 (9.6)	0 (0.0)	1 (5.9)	2 (5.4)	4 (5.7)	1 (11)
Personnel attitude	1 (1.2)	2 (12)	1 (5.9)	1 (2.7)	2 (2.9)	0 (0.0)
Hunch	14 (17)	3 (18)	3 (18)	9 (24)	10 (14)	2 (22)
Other	0 (0.0)	7 (41)	1 (5.9)	3 (8.1)	19 (27)	1 (1.1)
Pregnancy*	7 (8.3)	1 (5.9)	5 (29)	5 (14)	10 (14)	1 (1.1)
Missing	6	1	15	3	2	18

^*^Only applies to late answers

### Behaviours

There are two measurable behaviours which may indicate whether participants were aware of their trial allocation: timing of intercourse and occurrence of protocol violations.

#### Intercourse score

Intercourse scores were determined based on the timing of reported intercourse events relative to the most fertile window, as recorded in the study diary. The distribution of low, medium, and high scores was 1%, 10%, and 89% in the endometrial scratching group, and 4%, 7%, and 89% in the placebo group (*p* = 0.359) (Table 3). Unexpectedly, those who believed they were in the placebo group reported slightly more intercourse in both the endometrial scratching group (*p* = 0.079) and the placebo group (*p* = 0.073).

A total of 47 women committed protocol violations, with 24 in the endometrial scratching group and 23 in the placebo group, showing no significant difference between groups (*p* = 0.501). In the endometrial scratching group, women who guessed they were in the placebo group were more likely to commit a protocol violation compared to those who guessed placebo though this difference was not statistically significant (*p* = 0.115). In the placebo group, there was no evidence of an association between participant guesses and protocol violations (*p* = 0.897) (Table 3).

## Discussion

This study aimed to assess whether the use of a placebo procedure in an endometrial scratching trial effectively blinded participants to their assigned trial group. The results demonstrated most participants (56%) correctly guessed their allocation; the remainder either guessed incorrectly (32%) or were unsure (21%). James and Bang’s blinding indices both indicated probable lack of blinding, which was possibly more pronounced in the endometrial scratching arm. Pain and post-procedure bleeding were key factors contributing to unblinding in this study. Women in the endometrial scratching group reported higher pain scores and were more likely to experience post-procedure bleeding compared to those in the placebo group. Higher pain scores were also associated with a greater likelihood of guessing assignment to the endometrial scratching group.

The implication of the observed unblinding is unclear given that we did not detect problematic impact on participant behaviour, such as protocol violations or differences in intercourse patterns between groups. In fact, unexpectedly, we observed higher intercourse scores in those guessing they were in the placebo group. As these behaviours were largely self-reported by the participants, they are prone to bias such as social desirability bias, and it remains possible that the unblinding led to undetected changes to the behaviour of participants or personnel. However, we might expect any unblinding to favour the intervention arm, and the overall trial results found no evidence of an effect of endometrial scratching [23, 24]. These findings may challenge the assumption that similar intercourse behaviours across trial arms confirm blinding [26, 27]. Although we did not detect changes in intercourse behaviour or protocol violations because of unblinding, it is possible that other unmeasured factors, like stress, may still influence outcomes. Additionally, although the blinding indices suggest unblinding overall, half of participants did not guess their allocation correctly—which is still preferable to complete unblinding. However, it is because of the importance of the impact on behaviour rather than the blinding itself that the most recent CONSORT guidelines have removed the recommendation to report on blinding evaluation [28].

Researchers have debated the optimal timing for assessing participant blinding, with many suggesting that early guesses are more reliable, as later guesses may be influenced by knowledge of outcomes [12]. In our study, participants who conceived were more likely to guess they were in the scratch arm. However, overall, we observed little difference between early and late blinding guesses.

Our team and others have also performed large trials of endometrial scratching among participants undergoing IVF [29, 30]. In these studies, an open-label design was used—with no blinding of participants or personnel. The decision to forego a sham procedure was based on the assumption that IVF intervention occurs in the laboratory; all participants are highly motivated to conceive as they have one discrete opportunity to conceive from the IVF cycle, thereby reducing the opportunity for performance bias. However, in these IVF studies, we observed a higher incidence of protocol violations in the control arms, including control participants seeking endometrial scratching and participant withdrawal due to dissatisfaction with their allocation. Notably, a greater proportion of participants in the scratching arms proceeded to embryo transfer, suggesting that knowledge of allocation influenced participant or clinician decisions to cancel or defer the IVF cycle or embryo transfer, with more cancellations occurring in the control groups. This was unexpected given our assumption that blinding was not as important in the IVF context. We had anticipated more opportunity for performance bias in the studies where participants were trying to conceive from intercourse, where motivation to conceive may be increased by knowledge of allocation to a possible infertility therapy—e.g. participants in the scratch arm having more intercourse or better timing their sex in order to capitalise on their exposure to the scratch. All of our assumptions were proven incorrect—the procedure did not successfully blind people, couples in the placebo group had more intercourse than the intervention group, and we observed possible performance bias in the IVF study after deciding a sham procedure was not necessary.

The sham procedure used in this study involved placing the pipelle in the posterior fornix without cervical or uterine manipulation. This approach was intended to balance the objectives of achieving blinding without introducing contamination from a sham procedure that could cause some level of endometrial injury, and to minimise even small risks to participants, such as vasovagal response or infection. However, it resulted in significantly less pain and discomfort compared to the actual intervention. While complete blinding could theoretically be achieved by performing the procedure under general anaesthesia, ethical, medical, and financial considerations make this an impractical solution. Nevertheless, placebo-controlled surgical trials in gynaecology, particularly in the context of endometriosis, demonstrate that more invasive strategies to optimise blinding can be justified under carefully controlled conditions [31, 32]. More broadly, this challenge is not unique to the present study. Other gynaecological trials of procedural interventions have encountered similar difficulties in achieving effective blinding. For instance, in a recent RCT comparing carbon dioxide vaginal laser with a sham procedure, extensive efforts were undertaken to standardise the procedural experience across groups, including matching discomfort as well as auditory and visual cues, yet participants in the intervention arm reported lower procedural tolerance than those in the sham arm [33].

The decision to implement a sham procedure in an endometrial scratching trial involves multiple considerations beyond its effectiveness in blinding participants. On the one hand, a placebo procedure reduces protocol violations, such as control group participants seeking endometrial scratching independently. If unblinding occurs, it happens on the day of the procedure, limiting participants’ ability to seek out the study procedure or other off-protocol infertility therapy privately. For example, in the aforementioned IVF trials, we observed a small number of control participants (8/674) undergoing endometrial scratching privately [34]. Furthermore, blinding participants also enables blinding of trial and clinic staff, reducing possible bias in prioritising or pre-booking IVF for those receiving endometrial scratching.

On the other hand, a sham procedure may slow recruitment [35], as participants might decline additional clinic visits. Blinding can cause distress and tension between participants and trial staff. Though safe, the placebo procedure may cause some pain, require extra clinic visits, and add financial and time burdens on patients and clinics. One further and very minor disadvantage includes, logistically, only the performing clinician (and possibly a chaperone) should be aware of participant allocations, requiring additional administrative controls.

Limitations of this study included the small sample size and lack of a formal power calculation, which may have influenced the ability to detect unblinding. Blinding assessment was introduced 6 months after recruitment commenced; however, this still captured 87% of the overall trial population. Missing data was also a concern, as some participants did not complete self-reported outcomes, and 15 participants were excluded for not providing an allocation guess. Only one sham procedure was tested, and other approaches, such as pipelle placement at the internal os [36] or using an endometrial sound [37], may have different blinding effectiveness.

## Conclusion

Despite the use of a sham procedure, over half of participants in these randomised trials correctly guessed their treatment allocation, with pain and bleeding emerging as key factors contributing to unblinding. While blinding was not fully successful, we found no consistent evidence that this influenced participant behaviour, such as protocol violations or timing of intercourse—however, these were self-reported behaviours and there may be other important undetected changes. These findings highlight the challenges of achieving and interpreting blinding in trials of physical procedures. The feasibility and impact of blinding are likely to vary by context, even in different assisted reproductive technology trials, where procedural cues or patient expectations may influence both blinding and behaviour.

## Supplementary Information

Supplementary Material 1.

## Data Availability

The datasets used and/or analysed during the current study are available from the corresponding author on reasonable request.

## References

[1] Higgins JPT, Thomas J, Chandler J, Cumpston M, Li T, Page MJ, et al. (editors). Cochrane handbook for systematic reviews of interventions version 6.5 (updated 2024). Cochrane, 2024. Available from: https://www.cochrane.org/handbook.

[2] Wood L, Egger M, Gluud LL, Schulz KF, Jüni P, Altman DG, et al. Empirical evidence of bias in treatment effect estimates in controlled trials with different interventions and outcomes: meta-epidemiological study. BMJ. 2008;336(7644):601–5. 10.1136/bmj.39465.451748.AD PMC2267990 18316340

[3] Jüni P, Altman DG, Egger M. Assessing the quality of controlled clinical trials. BMJ. 2001;323(7303):42–6. 10.1136/bmj.323.7303.42 PMC1120670 11440947

[4] Moher D, Jones A, Cook DJ, Jadad AR, Moher M, Tugwell P, et al. Does quality of reports of randomised trials affect estimates of intervention efficacy reported in meta-analyses? Lancet. 1998;352(9128):609–13. 10.1016/S0140-6736(98)01085-X 9746022

[5] Page MJ, Higgins JP, Clayton G, Sterne JA, Hróbjartsson A, Savović J. Empirical evidence of study design biases in randomized trials: systematic review of meta-epidemiological studies. PLoS ONE. 2016;11(7):e0159267. 10.1371/journal.pone.0159267 PMC4939945 27398997

[6] Moustgaard H, Clayton GL, Jones HE, Boutron I, Jørgensen L, Laursen DR, et al. Impact of blinding on estimated treatment effects in randomised clinical trials: meta-epidemiological study. bmj. 2020;368:l6802. 10.1136/bmj.l6802 PMC7190062 31964641

[7] Hróbjartsson A, Emanuelsson F, Skou Thomsen AS, Hilden J, Brorson S. Bias due to lack of patient blinding in clinical trials. A systematic review of trials randomizing patients to blind and nonblind sub-studies. Int J Epidemiol. 2014;43(4):1272–83. 10.1093/ije/dyu115 PMC4258786 24881045

[8] Kahan BC, Rehal S, Cro S. Blinded outcome assessment was infrequently used and poorly reported in open trials. PLoS ONE. 2015;10(6):e0131926. 10.1371/journal.pone.0131926 PMC4488018 26120839

[9] Schulz KF, Grimes DA. Blinding in randomised trials: hiding who got what. Lancet. 2002;359(9307):696–700. 10.1016/S0140-6736(02)07816-9 11879884

[10] Onghena P. Resentful demoralization. Encyclopedia of statistics in behavioral science. 2005.

[11] Adamson J, Cockayne S, Puffer S, Torgerson DJ. Review of randomised trials using the post-randomised consent (Zelen’s) design. Contemp Clin Trials. 2006;27(4):305–19. 10.1016/j.cct.2005.11.003 16455306

[12] Fergusson D, Glass KC, Waring D, Shapiro S. Turning a blind eye: the success of blinding reported in a random sample of randomised, placebo controlled trials. BMJ. 2004;328(7437):432. 10.1136/bmj.37952.631667.EE PMC344259 14761905

[13] Hróbjartsson A, Forfang E, Haahr M, Als-Nielsen B, Brorson S. Blinded trials taken to the test: an analysis of randomized clinical trials that report tests for the success of blinding. Int J Epidemiol. 2007;36(3):654–63. 10.1093/ije/dym020 17440024

[14] Sackett DL. Turning a blind eye: why we don’t test for blindness at the end of our trials. BMJ. 2004;328(7448):1136. 10.1136/bmj.328.7448.1136-a PMC406365 15130997

[15] Bang H, Park JJ. Blinding in clinical trials: a practical approach. J Altern Complement Med. 2013;19(4):367–9. 10.1089/acm.2012.0210 23140113

[16] What to expect. Endometrial scratch. 2017. Available at: http://www.whattoexpect.com/forums/fertility-treatments/topic/endometrial-scratch-43.html.

[17] BabyCentre. Endometrial scratch. 2017. Available at: http://community.babycentre.co.uk/post/a24072687/endometrial_scratch.

[18] Rees JR, Wade TJ, Levy DA, Colford JM Jr, Hilton JF. Changes in beliefs identify unblinding in randomized controlled trials: a method to meet CONSORT guidelines. Contemp Clin Trials. 2005;26(1):25–37. 10.1016/j.cct.2004.11.020 15837450

[19] Bang H, Flaherty SP, Kolahi J, Park J. Blinding assessment in clinical trials: a review of statistical methods and a proposal of blinding assessment protocol. Clin Res Regul Aff. 2010;27(2):42–51.

[20] Orne MT. On the social psychology of the psychological experiment: with particular reference to demand characteristics and their implications. In: Sociological methods. Routledge; 2017. p. 279–99.

[21] James KE, Bloch DA, Lee KK, Kraemer HC, Fuller RK. An index for assessing blindness in a multi-centre clinical trial: disulfiram for alcohol cessation—a VA cooperative study. Stat Med. 1996;15(13):1421–34. 10.1002/(SICI)1097-0258(19960715)15:13<1421::AID-SIM266>3.0.CO;2-H 8841652

[22] Bang H, Ni L, Davis CE. Assessment of blinding in clinical trials. Control Clin Trials. 2004;25(2):143–56. 10.1016/j.cct.2003.10.016 15020033

[23] Wong TY, Lensen S, Wilkinson J, Glanville EJ, Acharya S, Clarke F, et al. Effect of endometrial scratching on unassisted conception for unexplained infertility: a randomized controlled trial. Fertil Steril. 2022;117(3):612–9. 10.1016/j.fertnstert.2021.12.009 35105443

[24] Glanville EJ, Wilkinson J, Sadler L, Wong TY, Acharya S, Aziz N, et al. A randomized trial of endometrial scratching in women with PCOS undergoing ovulation induction cycles. Reprod Biomed Online. 2022;44(2):316–23. 10.1016/j.rbmo.2021.10.008 34893436

[25] Kolahi J, Bang H, Park J. Towards a proposal for assessment of blinding success in clinical trials: up-to-date review. Community Dent Oral Epidemiol. 2009;37(6):477–84. 10.1111/j.1600-0528.2009.00494.x PMC3044082 19758415

[26] Brent K, Hadden WE, Weston-Webb M, Johnson NP. After the FLUSH trial: a prospective observational study of lipiodol flushing as an innovative treatment for unexplained and endometriosis-related infertility. Aust N Z J Obstet Gynaecol. 2006;46(4):293–7. 10.1111/j.1479-828X.2006.00595.x 16866789

[27] Johnson N, Farquhar C, Hadden W, Suckling J, Yu Y, Sadler L. The FLUSH trial—flushing with lipiodol for unexplained (and endometriosis-related) subfertility by hysterosalpingography: a randomized trial. Hum Reprod. 2004;19(9):2043–51. 10.1093/humrep/deh418 15271870

[28] Schulz KF, Altman DG, Moher D, Fergusson D. CONSORT 2010 changes and testing blindness in RCTs. Lancet. 2010;375(9721):1144–6. 10.1016/S0140-6736(10)60413-8 20338625

[29] Lensen S, Osavlyuk D, Armstrong S, Stadelmann C, Hennes A, Napier E, et al. A randomized trial of endometrial scratching before in vitro fertilization. N Engl J Med. 2019;380(4):325–34. 10.1056/NEJMoa1808737 30673547

[30] Van Hoogenhuijze N, Mol F, Laven J, Groenewoud E, Traas M, Janssen C, et al. Endometrial scratching in women with one failed IVF/ICSI cycle—outcomes of a randomised controlled trial (SCRaTCH). Hum Reprod. 2021;36(1):87–98. 10.1093/humrep/deaa268 PMC7801792 33289528

[31] Abbott J, Hawe J, Hunter D, Holmes M, Finn P, Garry R. Laparoscopic excision of endometriosis: a randomized, placebo-controlled trial. Fertil Steril. 2004;82(4):878–84. 10.1016/j.fertnstert.2004.03.046 15482763

[32] Mackenzie SC, Stephen J, Williams L, Daniels J, Norrie J, Becker CM, et al. Effectiveness of laparoscopic removal of isolated superficial peritoneal endometriosis for the management of chronic pelvic pain in women (ESPriT2): protocol for a multi-centre randomised controlled trial. Trials. 2023;24(1):425. 10.1186/s13063-023-07386-x PMC10286505 37349849

[33] Mension E, Alonso I, Anglès-Acedo S, Ros C, Otero J, Villarino Á, et al. Effect of fractional carbon dioxide vs sham laser on sexual function in survivors of breast cancer receiving aromatase inhibitors for genitourinary syndrome of menopause: the LIGHT randomized clinical trial. JAMA Netw Open. 2023;6(2):e2255697. 10.1001/jamanetworkopen.2022.55697 PMC9918877 36763359

[34] Lensen S, Martins W, Nastri C, Sadler L, Farquhar C. Pipelle for pregnancy (PIP): study protocols for three randomised controlled trials. Trials. 2016;17:1–14. 10.1186/s13063-016-1301-9 PMC4847336 27121007

[35] Hummer M, Holzmeister R, Kemmler G, Eder U, Hofer A, Kurzthaler I, et al. Attitudes of patients with schizophrenia toward placebo-controlled clinical trials. J Clin Psychiatry. 2003;64(3):277–81. 10.4088/jcp.v64n0308 12716268

[36] Parsanezhad ME, Dadras N, Maharlouei N, Neghahban L, Keramati P, Amini M. Pregnancy rate after endometrial injury in couples with unexplained infertility: a randomized clinical trial. Iran J Reprod Med. 2013;11(11):869. PMC3941397 24639710

[37] Gibreel A, El-Adawi N, Elgindy E, Al-Inany H, Allakany N, Tournaye H. Endometrial scratching for women with previous IVF failure undergoing IVF treatment. Gynecol Endocrinol. 2015;31(4):313–6. 10.3109/09513590.2014.994603 25561347

